# Whole Genome Sequencing, Antibiotic Resistance, and Epidemiology Features of Nontyphoidal *Salmonella* Isolated From Diarrheic Children: Evidence From North China

**DOI:** 10.3389/fmicb.2022.882647

**Published:** 2022-05-16

**Authors:** Wei Zhao, Xin Li, Xuening Shi, Kewei Li, Ben Shi, Jingyu Sun, Chao Zhao, Juan Wang

**Affiliations:** ^1^Jilin Center for Disease Prevention and Control, Changchun, China; ^2^School of Public Health, Jilin University, Changchun, China

**Keywords:** nontyphoidal *Salmonella*, children, antimicrobial resistance, WGS, risk assessment

## Abstract

Nontyphoidal *Salmonella* (NTS) in children remains a growing burden on public health and often causes children to be hospitalized with diarrheic symptoms. In this work, 260 strains of human *Salmonella* isolated from Jilin, China were characterized by serotypes and antimicrobial resistance using whole genome sequencing (WGS). The most prevalent serotype was *Salmonella enteritidis* (47.3%), followed by *S*. I 4,[5],12:i:- (33.1%), and *Salmonella Typhimurium* (7.3%). Furthermore, the consistency between resistance phenotype and genotype was confirmed. Similarly, strains harbored *bla*_*TEM*−1*B*_ and *tetA* genes were detected, which verified the level of resistant phenotype in β-lactams and tetracyclines. The presence of a single mutation in *parC, gyrA*, and *qnrS1* genes corresponding to quinolones was also observed. In our work, multilocus sequence typing (MLST) and core genome multilocus sequence typing (cgMLST) were found to have a high resolution to molecular traceability, and the combination of both was conducive to practical application in an actual situation. Taking all of this into account, we suggested that the comprehensive surveillance of *Salmonella* infection in children should be carried out to monitor antimicrobial-resistant trends from various sources and to alert on outbreaks of foodborne diseases to protect public health.

## Introduction

For a few years, nontyphoidal *Salmonella* (NTS) infection has been among the most common etiological causes of diarrheal and foodborne diseases (Balasubramanian et al., 2019). It is estimated that over 2.8 billion cases can be attributed to *Salmonella* infection. Children under 5 years old bear the burden of the major disease, especially in low-income regions (Havelaar et al., 2015; GBD 2017 Non-Typhoidal *Salmonella* invasive disease collaborators, 2019; Gilchrist and MacLennan, 2019). NTS generally causes self-limiting diarrheal illness, but may typically lead to severe invasive NTS for children's lack of immunity (Abuaita et al., 2021; Rana et al., 2021). Infection with different NTS serovars can present various epidemiological and clinical manifestations. Clinically, acute gastroenteritis with the onset of fever, vomiting, abdominal pains, and various diarrhea are the classic symptoms of NTS (Chen et al., 2013; Aoki et al., 2017). Antimicrobial agents may be required in the cases of invasive NTS (Crump et al., 2015). However, with the widespread use and abuse of antibiotics, especially quinolones and third-generation cephalosporins, drug-resistant bacteria can spread through antimicrobial-resistant genes (ARGs), and multiple drug-resistant (MDR) strains have constantly appeared (McDermott et al., 2018; Kariuki et al., 2019). Furthermore, the increasing rate of antimicrobial resistance poses a serious threat to healthcare systems and public health.

So far, over 2,500 *Salmonella* enteric serovars have been recognized, and *S. Enteritidis* and *S. Typhimurium* are the most prevalent serotypes (Michael and Schwarz, 2016; Jazeela et al., 2020). In recent years, emerging *Salmonella* serovars, such as *S*. I 4,[5],12:i:- have increased rapidly and spread globally. The traditional serotypes determined by a combination of O antigen and H antigens have some limitations. Currently, molecular typing technologies, such as multilocus sequence typing (MLST) and whole genome sequencing (WGS) have been applied due to their strong specificity, ease of operation, short detection time, and low cost. MLST has been widely used in epidemiology investigation because of its simplicity and repeatability. In addition, it can achieve network data sharing and comparison (Foley et al., 2006; Tang et al., 2019).

At the same time, with the decreasing cost of genome sequencing and the continuous advancement of bioinformation analysis technology, WGS is gradually applied to the outbreak investigation and epidemiological analysis at the forefront of disease prevention and control (Guinea et al., 2021; Papić et al., 2021). WGS-based methods, such as core genome multilocus sequence typing (cgMLST), include all core genes loci present in all given isolates. The complete analysis offers a high-resolution readily maintained and easily-shared database only using the same or similar online templates (Maiden et al., 2013).

Considering the important hazard of *Salmonella* in children, the widespread antibiotic resistance of *Salmonella*, and the absence of data from north China, the object of this study is to explore the serotypes, antimicrobial resistance, and presence of ARGs in the strains isolated from diarrheic children under 5 years old in Jilin Province. These analyses can be used for risk assessments of diarrheic children and for establishing prevention and control measures against foodborne diseases.

## Materials and Methods

### Sample Collection and *Salmonella* Serotyping

From 2014 to 2019, a total of 54,962 fecal and anal swab samples eligible were collected from 54,962 patients with diarrhea in 19 sentinel hospitals distributed in the Jilin Province, China. Out of 54,962 samples, 260 were positive for *Salmonella* for primary identification by sentinel hospitals. Then, further confirmation was conducted by Jilin Provincial Center for Disease Control and Prevention. First, according to the Chinese national standard GB4789.4 (2016), biochemical identification testing was performed by the Jilin Provincial Center for Disease Control and Prevention using API20E intestinal bacteria reagent identification strips (bioMerieux, France) for confirmation. Finally, 260 isolates were positive for *Salmonella* of 54,962 samples. First, raw data were assembled by splicing software SPAdes V3.13.1. Second, *Salmonella* serotyping was predicted by the genome assembly sequence using SeqSero2 1.2.1 with a software built-in database. Third, according to the Kauffmann–White scheme, serotypes were determined by serum agglutination testing using commercial antisera (SSI, Copenhagen, Denmark) for confirmation. *S. Typhimurium* ATCC14028 and *S. Enteritis* CMCC50035 were used for quality control.

### Whole Genome Sequencing

*Salmonella* strains were grown overnight at 37°C on Luria-Bertani (LB) agar, and the strains are listed in Supplementary Table 2. Genomic DNA was extracted using the DNeasy Blood & Tissue Kits (Qiagen, Germany) according to the manufacturer's protocol. Genomic DNA libraries were generated using the NEB NEXT ultra^TM^ DNA Library Prep Kit Illumina (NEB, USA). After PCR, the products were purified by the AMPure XP system (Beckman Coulter, Beverly, USA). DNA concentration was measured by the Qubit®3.0 Fluorometer (Invitrogen, USA). The libraries were sequenced on the Illumina NovaSeq6000 system. The obtained reads were trimmed with fqCleanerv.3.0 (Alexis Criscuolo, Institut Pasteur) and assembled using SPAdes v3.13.1 software.

### MLST, CgMLST, and Phylogenetic Analysis

BioNumerics 7.7 was used for analyzing sequence types (STs) according to the following traditional seven housekeeping genes *aroC, dnaN, hemD, hisD, purE, sucA*, and *thrA*, and sequence data of the isolates were extracted from their genome data. All cgMLST genes were downloaded from the PubMLST website (https://pubmlst.org/bigsdb?db=pubmlst_Salmonella_seqdef&page=downloadAlleles&tree=1). The loci in the genome sequences of each isolate were compared with the analysis template in Basic Local Alignment Search Tool (BLAST) with default settings, a threshold for similarity of 100% and a minimum length of 100%. Phylogenetic analysis was generated using BioNumerics 7.7 (www.applied-maths.com/download/software/).

### Antimicrobial Susceptibility Testing

The minimum inhibitory concentrations (MICs) of *Salmonella* isolates were determined by the broth dilution method following the Clinical and Laboratory Standards Institute guidelines CLSIM100ED31 (2021). Test antibiotics for *Salmonella* included ampicillin (AMP), ceftazidime (CAZ), ampicillin-sulbactam (AMS), imipenem (IMP), tetracycline (TET), nalidixic acid (NAL), cefoxitin (CFX), chloramphenicol (CHL), cefotaxime (CTX), cefazolin (CFZ), gentamicin (GEN), trimethoprim-sulfamethoxazole (SXT), azithromycin (AZM), and ciprofloxacin (CIP). *Escherichia coli* ATCC™ 25922 was used as a quality control strain.

### Antimicrobial Resistance Genes of *Salmonella*

Based on the genome annotation results in the Prokka 1.12, we compared the assembled genomes with ARGs databases, such as CARD (https://card.mcmaster.ca), ARDB (https://link.zhihu.com/?target=http%3A//ardb.cbcb.umd.edu/), and BacMet (http://bacmet.biomedicine.gu.se/) using ResFinder 4.0 software. We identified the presence of antimicrobial genes when the coverage was ≥70% and the identity was > 70%.

## Results

### Prevalence and Serotypes of NTS Isolated From Diarrheal Children

From to 2019, a total of 260 stool samples from diarrheal children were collected. Most of the samples were isolated in Jilin city (*n* = 87), while the lowest was from Siping city (*n* = 2). The distribution of *Salmonella* isolates across 9 cities of Jilin province is shown in Supplementary Table 3. In 260 isolated strains, *S. Enteritidis* accounted for 47.3% of all the collected isolates. *S*. I 4,[5],12:i:- (86/260) was second to the former. Abundant causative serovars, such as Agona, appeared in diarrheal children in various cities. The distribution of *Salmonella* serotypes with years is detailed in Supplementary Table 4.

### Epidemiology Features of NTS Infections

According to the data, 59.6% (155/260) of children were male, with the age ranging from 7 days to 60 months (Table 1). In total, 21.5% (56/260) of children were few months of age. The included children had a percentage of 13.5% (35/260) in hospitalization. In total, 22.7% (59/260) of these children were in treatment for acute mucoid diarrhea, and the remaining majority had watery stool, while the minority had diarrhea with visible blood. Abdominal pain was also relatively common in a series of symptoms (46/260). Other symptoms, such as fever (80/260) and vomiting (42/260), always appeared with the above-mentioned symptoms. No severe death was recorded. Suspected food consists of fruits and associated food, mixed food, egg and egg products, and animal products (Supplementary Table 1). However, most incidences may be attributed to unknown causes. One of the mainly suspicious exposed sources was fruits and mixed food. Egg and animal products also account for part of reason.

**Table 1:** Demographic and epidemiology features of diarrheal children infected with nontyphoidal *Salmonella* (NTS) (*n* = 260).

**Characteristic(s), symptoms, or outcome**	**Value (no. [%])**
**Gender**	
Male	155 (59.6)
Female	105 (40.4)
**Age**	
Age in months	56 (21.5)
Age of 1 to 5	204 (78.5)
**Clinical symptoms**	
Mucoid diarrhea	59 (22.7)
Blood diarrhea	7 (2.7)
Watery stool	164 (63.1)
Abdominal pain	46 (17.7)
Vomiting	42(16.2)
Fever (>37.5°C)	80 (30.8)
**Outcome**	
Inpatient	35 (13.5)
Outpatient	225 (86.5)
**Suspected exposed food**	
Fruits	68 (26.2)
Eggs and egg products	8 (3.1)
Mixed food	30 (11.5)
Animal products	17 (6.5)
Unknown food	137 (52.7)

### Antimicrobial Susceptibility

As shown in Table 2, high resistance to ampicillin (79.6%), ampicillin-sulbactam (47.7%), and cefazolin (45.8%) were predominated over other β-lactams. Correspondingly, nalidixic acid (60.8%) and tetracyclines (60.8%) showed higher resistance in quinolones and tetracyclines classes. Interestingly, none of the isolates were resistant to imipenem in this study. A total of 203 strains (78.1%) were resistant to three or more classes of antimicrobial agents and classified as MDR. Of 260 strains, 58 strains (22.3%) were resistant to five or more classes of antimicrobial agents. Only 5 strains (1.9%) were susceptible to all tested antimicrobial agents.

**Table 2:** The resistance of NTS isolates from diarrheal children in 2014–2019.

**Antimicrobial class**	**Antimicrobial agents**	**No. of strains (N/%)**					
		**Resistant (R)**		**Intermediate (I)**		**Susceptible (S)**	
Penicillins	Ampicillin (AMP)	207	79.6	1	0.4	52	20.0
β-lactam combination agents	Ampicillin-sulbactam (AMS)	124	47.7	78	30.0	58	22.3
Cephems	Cefotaxime (CTX)	37	14.2	4	1.5	219	84.2
	Ceftazidime (CAZ)	7	2.7	6	2.4	247	95.0
	Cefoxitin (CFX)	2	0.8	10	3.8	248	95.4
	Cefazolin (CFZ)	119	45.8	76	29.2	119	45.8
Sulfonamides	Trimethoprim-sulfamethoxazole (SXT)	66	25.4	-	-	194	74.6
Aminoglycosides	Gentamicin (GEN)	27	10.4	7	2.7	226	86.9
Quinolones	Nalidixic acid (NAL)	158	60.8	-	-	102	39.2
	Ciprofloxacin (CIP)	32	12.3	159	61.2	69	26.5
Phenicols	Chloramphenicol (CHL)	46	17.7	9	3.5	205	78.8
Carbapenems	Imipenem (IMP)	0	0	2	0.8	258	99.2
Tetracyclines	Tetracyclines (TET)	158	60.8	11	4.2	91	35.0
Macrolides	Azithromycin (AZM)	25	9.6	_	_	235	90.4
	Pansusceptible	5	1.9				
	≥1 antimicrobial class	255	98.1				
MDR	≥3 antimicrobial class	203	78.1				
	≥5 antimicrobial class	58	22.3				
	≥7 antimicrobial class	14	5.4				

### Occurrence of ARGs

According to the detailed results of drug resistance genes (Table 3), the predominant ARG confirmed was *aac (6')-Iaa* (99.6%), followed by *bla*_*TEM*−1*B*_ (63.1%), *sul2* (58.8%), and *aph (3”)-Ib* (53.5%). On the contrary, some genes, such as *tet G* (0.4%) and *mph A* (2.3%) were rarely identified. The other drug-resistance genes ranged from 4.6 to 53.5%. The predominant ARG identified in β-lactams resistant isolates was *bla*_*TEM*−1*B*_ (63.1%). Aminoglycoside class contained *aac(6')-Iaa, aph(6)-Id*, and *aph(3”)-Ib*. Sulfonamide class-resistant isolates were mainly positive for sul1, sul2, and sul3. Among the tetracycline class, *tet A, tet B, tet M*, and *tet G* genes were listed.

**Table 3:** Major antimicrobial genes and composition distribution in *Salmonella* strains.

**Antimicrobial classes**	**Antimicrobial resistance genes**	**Number of strains (%)**
β-Lactams	*bla_*TEM*−1*B*_*	63.1% (164/260)
	*bla_*OXA*−1_*	4.6% (12/260)
	*bla_*CTX*−*M*−55_*	2.7% (7/260)
	*bla_*CTX*−*M*−14_*	4.6%(12/260)
Sulfonamides	*sul1*	11.2% (29/260)
	*sul2*	58.8% (153/260)
	*sul3*	9.6% (25/260)
Aminoglycoside	*ac (6')-Iaa*	99.6%(259/260)
	*aph (6)-Id*	53.5%(139/260)
	*aph (3”)-Ib*	53.5% (139/260)
Phenicols	*floR*	11.9% (31/260)
	*cmlA1*	10% (26/260)
	*catB3*	4.6% (12/260)
Tetracyclines	*tet A*	17.3% (45/260)
	*tet B*	33.8% (88/260)
	*tet M*	5% (13/260)
	*tet G*	0.4% (1/260)
Quinolone	*qnrS1*	7.3% (19/260)
	*aac (6')-Ib-cr*	6.9% (18/260)
	*oqxA*	5.4% (14/260)
	*oqxB*	5.4% (14/260)
Macrolides	*mph(A)*	2.3% (6/260)

### MLST Analysis

**Figure 1:**
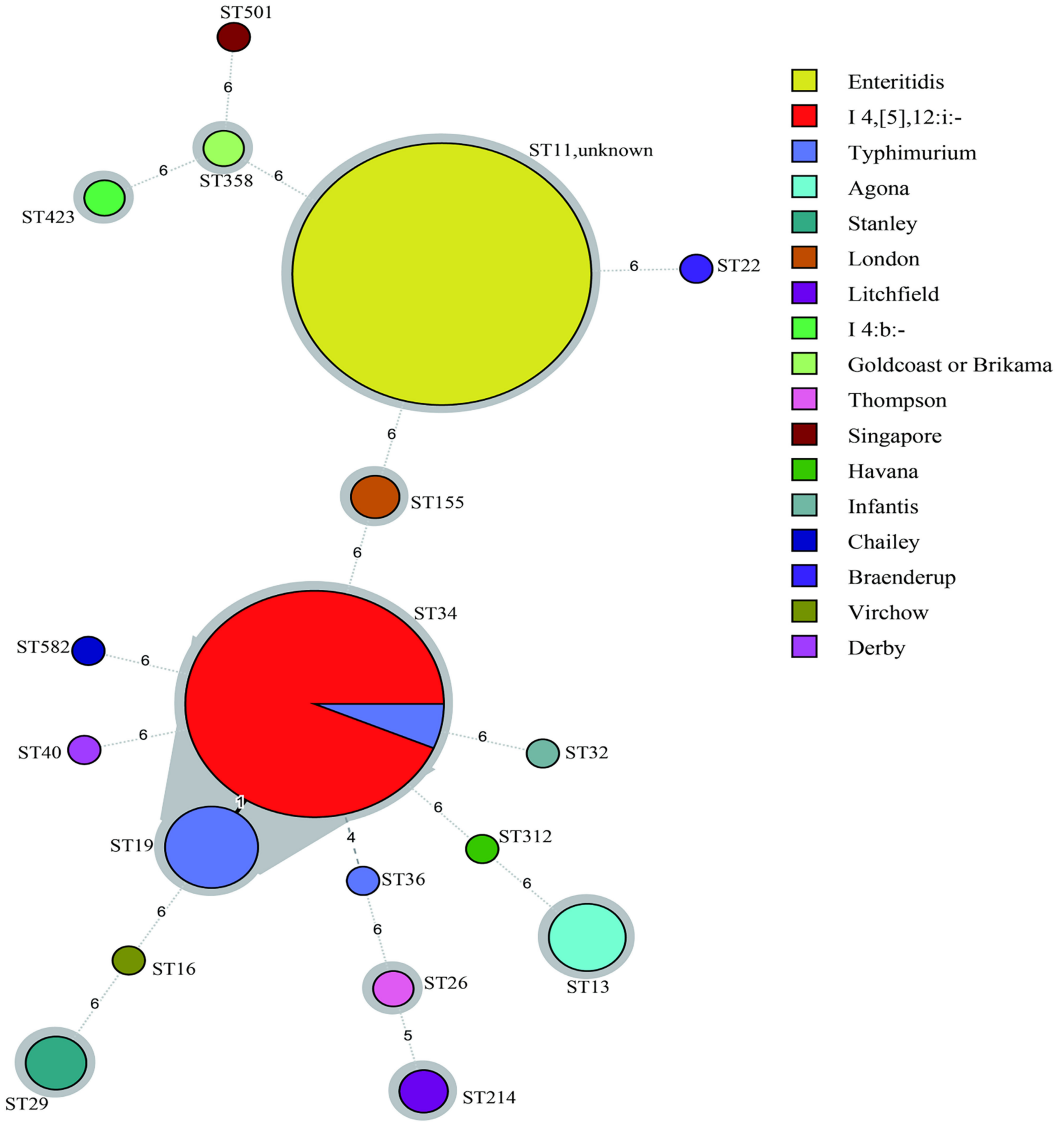
Minimum spanning tree (MST) of multilocus sequence typing (MLST) for 260 *Salmonella* strains. Allele distances among strains were labeled and STs were coded. The circle size represented the number of strains. The circle's color was filled according to serotypes and shades of gray indicated a close tie with strains.

According to the minimum spanning tree (MST) generated by serotypes (Figure 1), 18 international sequence types (STs) were identified in the 260 *Salmonella* strains and divided into 19 clusters. The dominant type was ST11 (113/260), followed by ST34 (91/260) and ST19 (12/260). However, 10 strains failed to classify into types. The MST based on cities of isolates is also constructed in Supplementary Figure 1. In this study, the results of MLST and serotyping from 260 isolates of patients with diarrhea in Jilin Province exhibited that these two types have good correspondence, except ST34. However, as shown in Figure 1, S. Enteritidis (ST11), S. Typhimurium (ST19/ST34), S. I 4,[5],12:i:- (ST34), S. Agona (ST13), S. London (ST155), S. Stanley (ST29), S. Litchfield (ST214), S. I 4:b:- (ST423), S. Stanley (ST29), and S. Thompson (ST26) showed associated profiles.

### CgMLST Analysis and Phylogenetic Analysis

**Figure 2:**
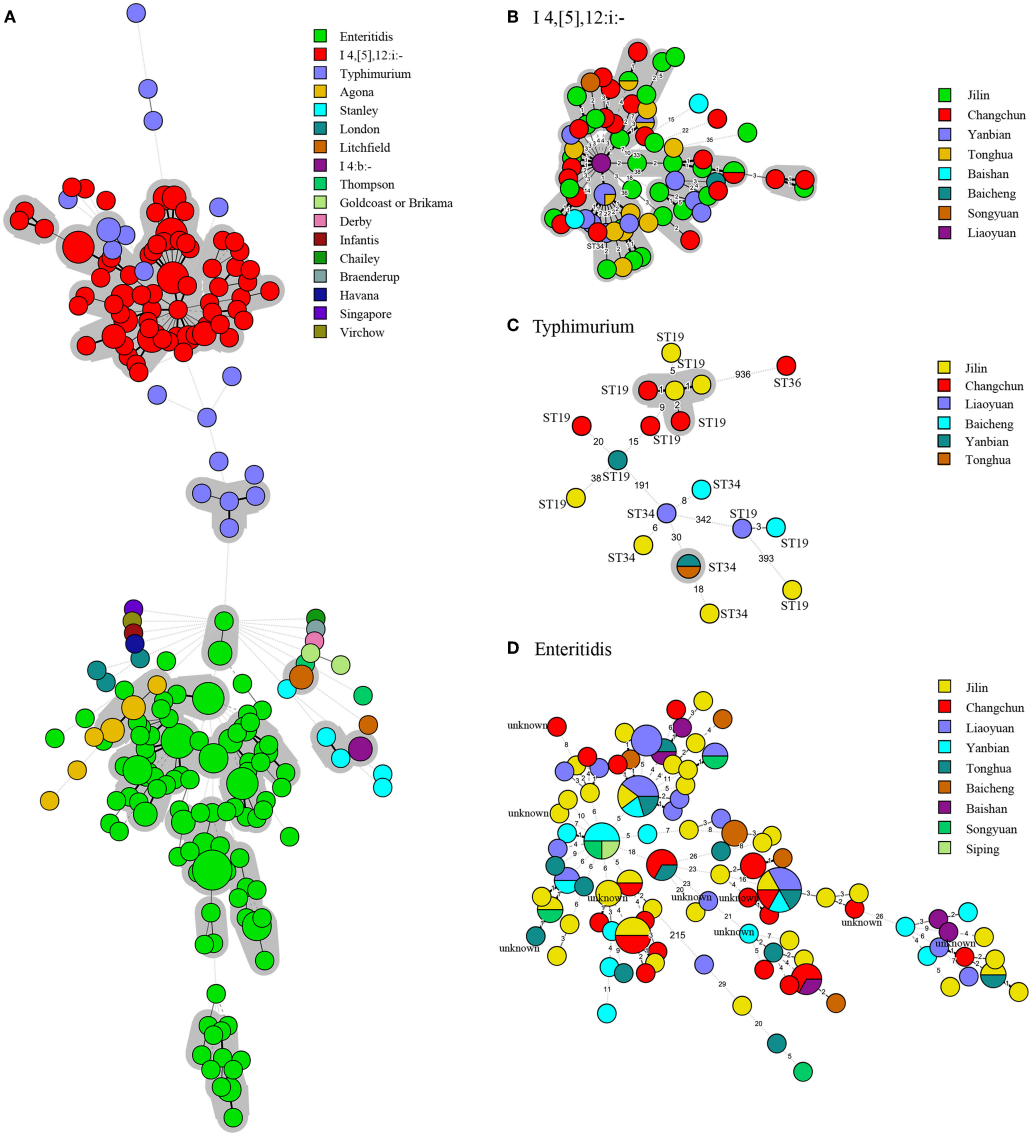
Minimum spanning tree of core genome MLST for 260 *Salmonella* strains. Every strain was filled with different colors according to its serotypes. Allele distances among B, C and D strains were labeled. The circle size represented the number of strains. **(A)** In total, 260 *Salmonella* strains MST based on core genome MLST (cgMLST). **(B)** The upper layers filled with red *Salmonella* strains showed the same STs of 34. **(C)** The middle scatter *Salmonella* strains with no serotype distribution law. **(D)** The lower layer filled with green *Salmonella* strains was all ST11.

**Figure 3:**
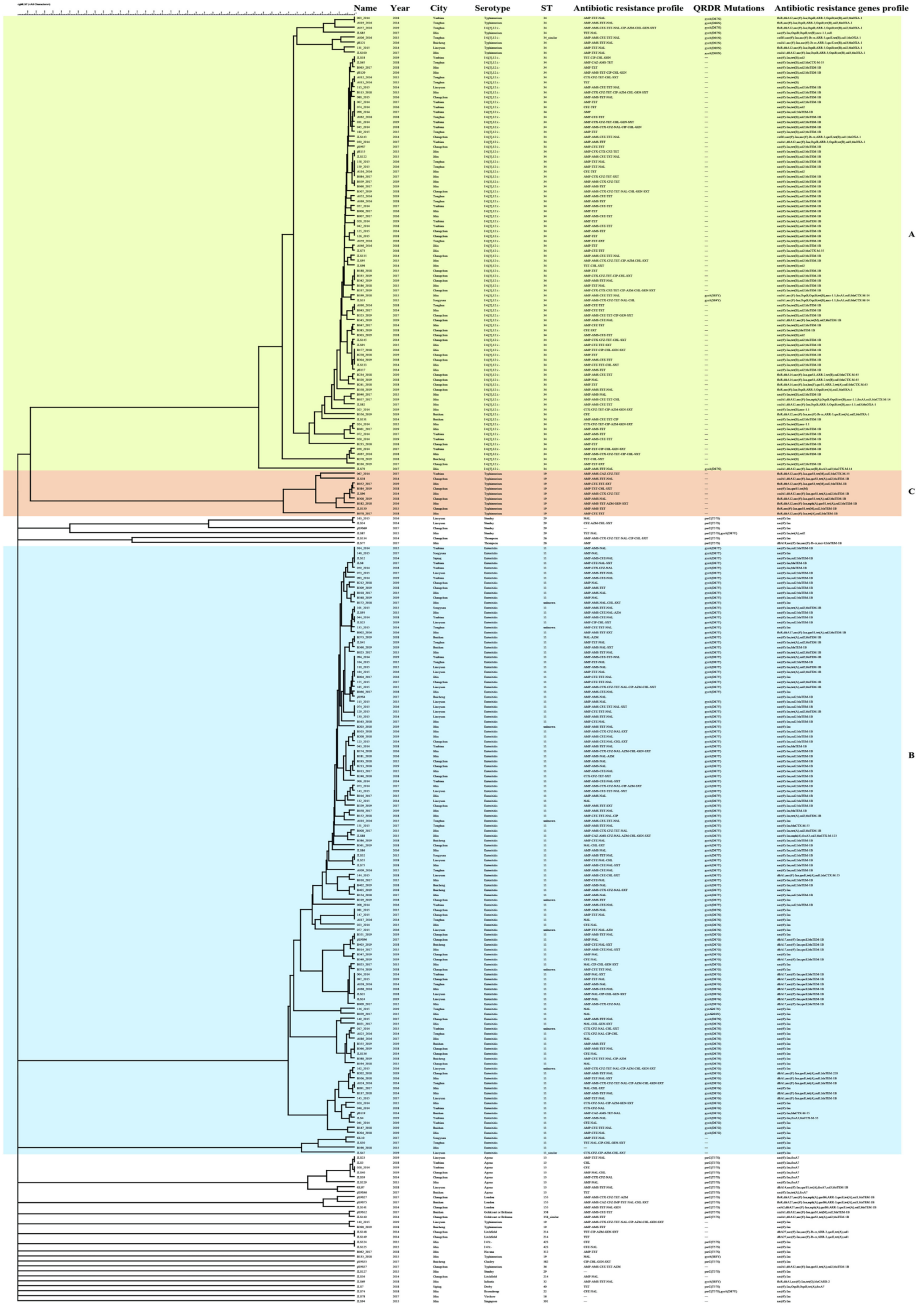
Phylogenetic analysis of 260 *Salmonella* strains from diarrheic children. The entries were arranged above the picture. Isolates, such as 003_2014 and A019_2016 with no allelic differences could be identified as the same clone. Discrepancies of alleles exceeding the scale above were considered unrelated. The dendrogram mainly contains A, B, and C three parts, corresponding to 92 *S*.I 4,[5],12:i:- and *Salmonella* Typhimurium strains, 123 *Salmonella* Enteritidis strains, and 9 *S*. Typhimurium isolates. The similarity index was labeled over the clusters to show the genetic relationships.

An MST analysis of 260 *Salmonella* strains was based on serotypes and cities of cgMLST (Figure 2 and Supplementary Figure 2). Compared with the MST analysis based on MLST, more detailed and intuitive associations of serotypes were displayed on cgMLST and mainly divided into B, C, and D parts. The B and D clusters showed the same STs. However, an apparent connection classified based on the cities was still not observed. A phylogenetic analysis based on cgMLST of 260 *Salmonella* strains is presented in Figure 3. The phylogenetic dendrogram included name, year, city, serotypes, STs, antimicrobial resistance profiles, and antimicrobials resistance genes profiles. In total, 260 *Salmonella* strains were defined as 208 tiny clusters that were in accordance with STs arrangement. The isolates were mainly grouped into three clusters (denoted as A, B, and C). Cluster A contained 92 isolates, such as S.I 4,[5],12:i:- and S. Typhimurium (Supplementary Figure 3), and they showed similar indexes ranging from 57.2 to 100%. Cluster B was the most in number and composed of 123 *S*. Enteritidis strains with a similar index of at least 60.7% (Supplementary Figure 4). Only 9 *S*. Typhimurium isolates were included in cluster C, and they presented nine patterns with a minimum similarity index of 77.7% (Supplementary Figure 5).

## Discussion

Our study combined clinical epidemiology and genomic data to depict the characteristics of NTS infections in northern China. In line with recent findings (Li et al., 2014; Zhan et al., 2019), *S*. Enteritidis and *S*. I 4,[5],12:i:- are the leading *Salmonella* serovars. *S*.I 4,[5],12:i:- currently represents the common serotype among human cases in many countries (Seixas et al., 2018; He et al., 2020). This serovar first appeared in the 1990s but increased considerably in the last 10 years, especially in Europe and Asia (Kawakami et al., 2019). The appearance of *S*. I 4,[5],12:i:- was always associated with foodborne disease outbreaks and accompanied by the MDR strains (Mølbak et al., 2014; Elnekave et al., 2018). Therefore, monitoring trends in various serovars over time and developing public health methods may help in focusing on preventative strategies. Our study has made a detailed epidemiological description based on symptoms of diarrhea cases. However, due to no certain evidence of suspected food and *Salmonella* diarrheas onset, the exact exposed food was missing. It was also impossible to identify the possible sources of infection for every sporadic case.

The emergence of *Salmonella* infections with MDR has become a major threat to public health worldwide. In our study, 203 (78.1%) isolated NTS were classified as MDR among 260 strains, and resistance occurred to antibiotics commonly used in treatment. A similar incidence rate was presented in studies by Duong et al. (2020) . In addition, four strains from children under the age of 1 were resistant to at least seven antimicrobials, and these results highlighted the need for effective surveillance of antimicrobials management in China. At present, the third generation cephalosporins antimicrobials are the new preferred antimicrobial therapy instead of quinolones in clinical trials for *Salmonella* infection (Kariuki et al., 2015). Different from the high resistance of quinolones, 14.2 and 2.7% resistant rates were observed in cefotaxime and ceftazidime, respectively. Similarly, a relatively low resistant rate was also observed in Brazil (Viana et al., 2020) and Greece (Mellou et al., 2021).

The consistency between genomic and phenotypic investigations has been successfully verified in our study. Resistance genes, such as *bla*_*TEM*−1*B*_, *bla*_*OXA*−1_, *bla*_*CTX*−*M*−55_, and *bla*_*CTX*−*M*−14_ that belonged to β-lactams were detected. The highest frequency of *bla*_*TEM*−1*B*_ of strains was consistent with studies in eastern China (Zheng et al., 2021). Extended-spectrum β-lactamases (ESBLs) *bla*_*CTX*−*M*−55_ were also detected. The carbapenems are the critical “last resort” antimicrobials to *Salmonella* infections for their stability toward ESBLs (Fernández et al., 2018), and in our study, the strains remain susceptible in phenotype and genotype.

According to the data (Hopkins et al., 2005; Zhan et al., 2019), mutations in chromosome and plasmid-mediated resistance genes all contributed to quinolones resistance, which is commonly used quinolones for *Salmonellosis*. Similar to a previous report in Taiwan (Fang et al., 2021), mutations in *parC* and *gyrA* were more likely to cause the resistance of quinolones.

Moreover, resistance to quinolones can be acquired by plasmid-mediated quinolone resistance (PMQR) genes, such as *oqxAB*. As shown in Supplementary Table 5, 28 of 170 quinolones resistance strains harbored PMQR genes. As for plasmid-mediated genes, the *qnrS1* gene was also detected in retail food (Yang et al., 2020), which may prove the horizontal transfer of plasmids. Previous studies reported that quinolones were strongly linked to the overuse of antibiotics in food animals (de Jong et al., 2012; Redgrave et al., 2014). Meanwhile, quinolones have just been out of use in food-producing animals in China since 2018 (Cuypers et al., 2018). Moreover, previous findings also described quinolones as therapy producing resistance to food and water-borne infections (Threlfall, 2002; Molina et al., 2016). Hence, further efforts are warranted to control the spread of resistance trends from multiple sources. In our study, we found that *bla*_*TEM*−1*B*_ and *oxqAB* coexisted in four MDR strains. This suggested that the common existence of these genes leads to resistance to both quinolone and β-lactams. Further efforts to explore the transmission and influence of multiply classes of antibiotics coexistence will be of interest.

Multilocus sequence typing was used for the epidemiology investigation of NTS in our study. ST11 and ST34 were the predominant types, which was in line with the distribution of animal farms and retailed foods in China (Yang et al., 2019; Li et al., 2020). These findings support the widespread transmission of *Salmonella* strains among different regions. MLST and cgMLST showed a high level of molecular traceability. The tree diagrams based on MLST and cgMLST indicated that human *Salmonella* cases with unrelated epidemiological backgrounds were apt to be sporadic (Tönnies et al., 2021). In this study, consistency between MLST sequence type and phenotypic serotype was found to provide evidence for the genetic structure of *Salmonella*. However, 13 strains were not distinguished. It indicates that MLST cannot distinguish all isolates. Compared with MLST, cgMLST based on WGS presented a relatively high resolution, but more equipment and time were necessary. The combination of the two types can provide the origin and evolution basis of *Salmonella* strains and reveal the evolution and variation.

## Conclusion

In this article, we analyzed the epidemiological and molecular characteristics of NTS isolated from diarrheic children. Besides, *S*. Typhimurium and *S*. I 4,[5],12:i:- were the dominant serotypes. A considerable increasing AMR prevalence rate was observed, providing evidence between resistant phenotype and genotype. MLST and cgMLST were performed for serotype clustering, while cgMLST had high resolution and good repeatability. In conclusion, our study showed the genetic diversity of NTS isolates from diarrheic children and suggested the monitoring of antibiotic resistance and dominant STs of *Salmonella* in children.

## Data Availability Statement

The datasets presented in this study can be found in online repositories. The names of the repository/repositories and accession number(s) can be found below: NCBI - PRJNA817369.

## Author Contributions

WZ, CZ, and JW: conceptualization. KL, BS, and JS: experiment investigation. XL, XS, and WZ: writing, original draft preparation, and revising. JW provided critical comments on this work. All authors have read and agreed to the manuscript.

## Funding

The authors thank the funding support from the National Natural Science Foundation of China (grant number 82073603) and the Jilin Province Science and Technology Development Plan Item (grant number 20210204139YY).

## Conflict of Interest

The authors declare that the research was conducted in the absence of any commercial or financial relationships that could be construed as a potential conflict of interest.

## Publisher's Note

All claims expressed in this article are solely those of the authors and do not necessarily represent those of their affiliated organizations, or those of the publisher, the editors and the reviewers. Any product that may be evaluated in this article, or claim that may be made by its manufacturer, is not guaranteed or endorsed by the publisher.
