# Supplementary material for: Whole Genome Sequencing, Antibiotic Resistance, and Epidemiology Features of Nontyphoidal Salmonella Isolated From Diarrheic Children: Evidence From North China
Source: Front Microbiol. 2022 May 16;13:882647. doi: 10.3389/fmicb.2022.882647 (PMC9150820; doi:10.3389/fmicb.2022.882647)
Supplement: Supplementary file 1 [file Table_1.docx]

Supplementary Materials

**Whole genome sequencing, antibiotic resistance, and epidemiology features of nontyphoidal *Salmonella* isolated from diarrheic children: evidence from north China**

Wei Zhao *^a^, Xin Li ^b^, Xuening Shi ^b^, Kewei Li ^a^, Ben Shi ^a^, Jingyu Sun ^a^, Chao Zhao ^b^, Juan Wang *^b^

a. Jilin Center for Disease Prevention and Control, Changchun 130062, Changchun, China

b. School of Public Health, Jilin University, Changchun 130021, China

***Corresponding author:***Email: [weizhao81226@126.com](mailto:weizhao81226@126.com) ; [jwang0723@jlu.edu.cn](mailto:jwang0723@jlu.edu.cn)

**Table S2 The basic information of 260 *Salmonella* strains**

| Strain name | Specimen type | City | Year |
| --- | --- | --- | --- |
| B154_2018 | Stool | Jilin | 2018 |
| 057_2014 | Stool | Yanbian | 2014 |
| 056_2014 | Stool | Yanbian | 2014 |
| B084_2017 | Stool | Jilin | 2017 |
| A024_2016 | Stool | Tonghua | 2016 |
| JLS47 | Stool | Liaoyuan | 2015 |
| 165_2015 | Stool | Liaoyuan | 2015 |
| 164_2015 | Stool | Liaoyuan | 2015 |
| 163_2015 | Stool | Liaoyuan | 2015 |
| 162_2015 | Stool | Liaoyuan | 2015 |
| B355_2019 | Stool | Baishan | 2019 |
| B356_2019 | Stool | Baishan | 2019 |
| B351_2019 | Stool | Changchun | 2019 |
| B349_2019 | Stool | Changchun | 2019 |
| B013_2017 | Stool | Jilin | 2017 |
| jl19S69 | Stool | Changchun | 2019 |
| B303_2019 | Stool | Changchun | 2019 |
| B212_2018 | Stool | Changchun | 2018 |
| JLS136 | Stool | Changchun | 2018 |
| B194_2018 | Stool | Changchun | 2018 |
| B193_2018 | Stool | Changchun | 2018 |
| JLS122 | Stool | Jilin | 2018 |
| B152_2018 | Stool | Jilin | 2018 |
| JLS91 | Stool | Jilin | 2017 |
| B078_2017 | Stool | Jilin | 2017 |
| B040_2017 | Stool | Jilin | 2017 |
| JLS77 | Stool | Jilin | 2017 |
| B014_2017 | Stool | Jilin | 2017 |
| B006_2017 | Stool | Jilin | 2017 |
| A069_2016 | Stool | Tonghua | 2016 |
| A068_2016 | Stool | Jilin | 2016 |
| A052_2016 | Stool | Tonghua | 2016 |
| A057_2016 | Stool | Tonghua | 2016 |
| JLS55 | Stool | Tonghua | 2015 |
| jl19S75 | Stool | Baishan | 2019 |
| B361_2019 | Stool | Changchun | 2019 |
| jl19S4 | Stool | Baicheng | 2019 |
| B066_2017 | Stool | Jilin | 2017 |
| 151_2015 | Stool | Tonghua | 2015 |
| 159_2015 | Stool | Tonghua | 2015 |
| 158_2015 | Stool | Tonghua | 2015 |
| 155_2015 | Stool | Changchun | 2015 |
| 093_2014 | Stool | Yanbian | 2014 |
| 094_2014 | Stool | Yanbian | 2014 |
| 098_2014 | Stool | Yanbian | 2014 |
| B163_2018 | Anal swab | Jilin | 2018 |
| 180_2015 | Anal swab | Tonghua | 2015 |
| JLS7 | Stool | Siping | 2014 |
| 048_2014 | Anal swab | Yanbian | 2014 |
| 050_2014 | Stool | Yanbian | 2014 |
| JLS8 | Anal swab | Yanbian | 2014 |
| jl19S55 | Stool | Baicheng | 2019 |
| B373_2019 | Stool | Baishan | 2019 |
| B358_2019 | Stool | Changchun | 2019 |
| JLS130 | Stool | Changchun | 2018 |
| JLS79 | Stool | Jilin | 2017 |
| B021_2017 | Stool | Jilin | 2017 |
| B018_2017 | Stool | Jilin | 2017 |
| JLS78 | Stool | Jilin | 2017 |
| B016_2017 | Stool | Jilin | 2017 |
| JLS73 | Stool | Jilin | 2017 |
| B002_2016 | Stool | Jilin | 2016 |
| A086_2016 | Stool | Jilin | 2016 |
| A067_2016 | Stool | Jilin | 2016 |
| A066_2016 | Stool | Jilin | 2016 |
| JLS65 | Stool | Tonghua | 2016 |
| 149_2015 | Stool | Changchun | 2015 |
| 147_2015 | Stool | Changchun | 2015 |
| 146_2015 | Stool | Songyuan | 2015 |
| 145_2015 | Stool | Tonghua | 2015 |
| 144_2015 | Stool | Liaoyuan | 2015 |
| 143_2015 | Stool | Liaoyuan | 2015 |
| 140_2015 | Stool | Liaoyuan | 2015 |
| 044_2014 | Stool | Yanbian | 2014 |
| 045_2014 | Stool | Yanbian | 2014 |
| 043_2014 | Stool | Yanbian | 2014 |
| 041_2014 | Stool | Yanbian | 2014 |
| 042_2014 | Stool | Yanbian | 2014 |
| B309_2019 | Stool | Changchun | 2019 |
| B308_2019 | Stool | Changchun | 2019 |
| JLS140 | Stool | Changchun | 2018 |
| B202_2018 | Stool | Changchun | 2018 |
| JLS141 | Stool | Changchun | 2018 |
| JLS131 | Stool | Baishan | 2018 |
| B199_2018 | Stool | Jilin | 2018 |
| JLS127 | Stool | Jilin | 2018 |
| B172_2018 | Stool | Jilin | 2018 |
| B057_2017 | Stool | Jilin | 2017 |
| JLS88 | Stool | Jilin | 2017 |
| A116_2016 | Stool | Jilin | 2016 |
| JLS69 | Stool | Jilin | 2016 |
| A038_2016 | Stool | Tonghua | 2016 |
| A010_2016 | Stool | Tonghua | 2016 |
| JLS40 | Stool | Changchun | 2015 |
| JLS39 | Stool | Changchun | 2015 |
| 126_2015 | Stool | Changchun | 2015 |
| 125_2015 | Stool | Changchun | 2015 |
| JLS38 | Stool | Changchun | 2015 |
| 123_2015 | Stool | Changchun | 2015 |
| JLS36 | Stool | Changchun | 2015 |
| JLS33 | Stool | Songyuan | 2015 |
| JLS32 | Stool | Songyuan | 2015 |
| JLS34 | Stool | Tonghua | 2015 |
| 115_2015 | Stool | Tonghua | 2015 |
| 113_2015 | Stool | Liaoyuan | 2015 |
| 067_2014 | Stool | Yanbian | 2014 |
| JLS12 | Stool | Siping | 2014 |
| B360_2019 | Stool | Baishan | 2019 |
| B336_2019 | Stool | Changchun | 2019 |
| B335_2019 | Stool | Changchun | 2019 |
| B329_2019 | Stool | Changchun | 2019 |
| B323_2019 | Stool | Changchun | 2019 |
| B319_2019 | Stool | Changchun | 2019 |
| B188_2018 | Stool | Changchun | 2018 |
| B190_2018 | Stool | Jilin | 2018 |
| B186_2018 | Stool | Jilin | 2018 |
| JLS74 | Stool | Jilin | 2017 |
| A017_2016 | Stool | Tonghua | 2016 |
| A015_2016 | Stool | Tonghua | 2016 |
| A012_2016 | Stool | Tonghua | 2016 |
| 097_2015 | Stool | Changchun | 2015 |
| JLS25 | Stool | Liaoyuan | 2015 |
| JLS18 | Stool | Yanbian | 2014 |
| jl19S96 | Stool | Changchun | 2019 |
| B135_2018 | Stool | Jilin | 2018 |
| JLS85 | Stool | Jilin | 2017 |
| B043_2017 | Stool | Jilin | 2017 |
| B007_2017 | Stool | Jilin | 2017 |
| A062_2016 | Stool | Tonghua | 2016 |
| A060_2016 | Stool | Tonghua | 2016 |
| A059_2016 | Stool | Tonghua | 2016 |
| A019_2016 | Stool | Tonghua | 2016 |
| 104_2015 | Stool | Tonghua | 2015 |
| JLS30 | Stool | Tonghua | 2015 |
| 101_2015 | Stool | Songyuan | 2015 |
| GL10 | Stool | Songyuan | 2015 |
| 110_2015 | Stool | Liaoyuan | 2015 |
| JLS24 | Stool | Liaoyuan | 2015 |
| B402_2019 | Stool | Baicheng | 2019 |
| B403_2019 | Stool | Baicheng | 2019 |
| B405_2019 | Stool | Baicheng | 2019 |
| jl19S46 | Stool | Baishan | 2019 |
| B347_2019 | Stool | Changchun | 2019 |
| B345_2019 | Stool | Changchun | 2019 |
| B342_2019 | Stool | Changchun | 2019 |
| B348_2019 | Stool | Changchun | 2019 |
| B228_2018 | Stool | Changchun | 2018 |
| JLS143 | Stool | Changchun | 2018 |
| B235_2018 | Stool | Changchun | 2018 |
| B234_2018 | Stool | Changchun | 2018 |
| JLS145 | Stool | Changchun | 2018 |
| B182_2018 | Stool | Jilin | 2018 |
| B181_2018 | Stool | Jilin | 2018 |
| JLS129 | Stool | Jilin | 2018 |
| B177_2018 | Stool | Jilin | 2018 |
| B174_2018 | Stool | Jilin | 2018 |
| B091_2017 | Stool | Jilin | 2017 |
| JLS96 | Stool | Jilin | 2017 |
| JLS95 | Stool | Jilin | 2017 |
| JLS87 | Stool | Jilin | 2017 |
| JLS75 | Stool | Jilin | 2017 |
| B390_2019 | Stool | Baicheng | 2019 |
| B388_2019 | Stool | Baicheng | 2019 |
| B386_2019 | Stool | Baicheng | 2019 |
| B385_2019 | Stool | Changchun | 2019 |
| B384_2019 | Stool | Changchun | 2019 |
| B320_2019 | Stool | Changchun | 2019 |
| jl19S27 | Stool | Changchun | 2019 |
| B307_2019 | Stool | Changchun | 2019 |
| B304_2019 | Stool | Changchun | 2019 |
| B241_2018 | Stool | Changchun | 2019 |
| JLS149 | Stool | Changchun | 2018 |
| JLS148 | Stool | Changchun | 2018 |
| JLS135 | Stool | Changchun | 2018 |
| B208_2018 | Stool | Jilin | 2018 |
| JLS134 | Stool | Changchun | 2018 |
| JLS133 | Stool | Jilin | 2018 |
| B204_2018 | Stool | Jilin | 2018 |
| B203_2018 | Stool | Jilin | 2018 |
| JLS125 | Stool | Jilin | 2018 |
| JLS124 | Stool | Jilin | 2018 |
| B106_2018 | Stool | Jilin | 2018 |
| JLS100 | Stool | Jilin | 2018 |
| B103_2018 | Stool | Jilin | 2018 |
| JLS98 | Stool | Jilin | 2018 |
| B063_2017 | Stool | Jilin | 2017 |
| B062_2017 | Stool | Jilin | 2017 |
| B061_2017 | Stool | Jilin | 2017 |
| B060_2017 | Stool | Jilin | 2017 |
| B059_2017 | Stool | Jilin | 2017 |
| B047_2017 | Stool | Jilin | 2017 |
| JLS82 | Stool | Jilin | 2017 |
| B029_2017 | Stool | Jilin | 2017 |
| B010_2017 | Stool | Jilin | 2017 |
| B009_2017 | Stool | Jilin | 2017 |
| B008_2017 | Stool | Jilin | 2017 |
| B004_2017 | Stool | Jilin | 2017 |
| JLS41 | Stool | Tonghua | 2015 |
| 074_2014 | Stool | Yanbian | 2014 |
| 030_2014 | Stool | Yanbian | 2014 |
| 031_2014 | Stool | Yanbian | 2014 |
| A009_2016 | Stool | Tonghua | 2016 |
| A006_2016 | Stool | Tonghua | 2016 |
| 136_2015 | Stool | Tonghua | 2015 |
| 135_2015 | Stool | Tonghua | 2015 |
| 132_2015 | Stool | Liaoyuan | 2015 |
| 131_2015 | Stool | Liaoyuan | 2015 |
| 130_2015 | Stool | Liaoyuan | 2015 |
| 129_2015 | Stool | Liaoyuan | 2015 |
| JLS4 | Stool | Yanbian | 2014 |
| JLS3 | Stool | Yanbian | 2014 |
| 026_2014 | Stool | Yanbian | 2014 |
| 072_2014 | Stool | Yanbian | 2014 |
| 028_2014 | Stool | Yanbian | 2014 |
| 029_2014 | Stool | Yanbian | 2014 |
| 073_2014 | Stool | Jilin | 2014 |
| 024_2014 | Stool | Jilin | 2014 |
| 025_2014 | Stool | Jilin | 2014 |
| 020_2014 | Stool | Jilin | 2014 |
| 023_2014 | Stool | Jilin | 2014 |
| B366_2019 | Stool | Changchun | 2019 |
| B213_2018 | Stool | Changchun | 2018 |
| B337_2019 | Stool | Changchun | 2019 |
| jl19S57 | Stool | Changchun | 2019 |
| B240_2018 | Stool | Changchun | 2019 |
| B115_2018 | Stool | Jilin | 2018 |
| B058_2017 | Stool | Jilin | 2017 |
| JLS21 | Stool | Liaoyuan | 2015 |
| JLS23 | Stool | Liaoyuan | 2015 |
| 006_2014 | Stool | Yanbian | 2014 |
| 008_2014 | Stool | Yanbian | 2014 |
| 007_2014 | Stool | Yanbian | 2014 |
| 004_2014 | Stool | Yanbian | 2014 |
| jl19S7 | Stool | Changchun | 2019 |
| B374_2019 | Stool | Changchun | 2019 |
| jl19S12 | Stool | Baishan | 2019 |
| B378_2019 | Stool | Baicheng | 2019 |
| jlS121 | Stool | Baicheng | 2018 |
| jlS120 | Stool | Jilin | 2018 |
| B147_2018 | Stool | Baishan | 2018 |
| jlS119 | Stool | Baishan | 2018 |
| jlS115 | Stool | Jilin | 2018 |
| jlS117 | Stool | Jilin | 2018 |
| B137_2018 | Stool | Jilin | 2018 |
| JLS94 | Stool | Jilin | 2017 |
| JLS93 | Stool | Jilin | 2017 |
| B052_2017 | Stool | Jilin | 2017 |
| JLS86 | Stool | Jilin | 2017 |
| B025_2017 | Stool | Jilin | 2017 |
| B023_2017 | Stool | Jilin | 2017 |
| A025_2016 | Stool | Tonghua | 2016 |
| 081_2015 | Stool | Changchun | 2015 |
| 080_2015 | Stool | Changchun | 2015 |
| GL07 | Stool | Tonghua | 2015 |
| 077_2015 | Stool | Tonghua | 2015 |
| 074_2015 | Stool | Liaoyuan | 2015 |
| 073_2015 | Stool | Liaoyuan | 2015 |
| 003_2014 | Stool | Yanbian | 2014 |
| 014_2014 | Stool | Yanbian | 2014 |
| 017_2014 | Stool | Yanbian | 2014 |

**Table S3** Distribution of human *Salmonella* isolates by region (2014–2019)

| Year | | | | | | | |
| --- | --- | --- | --- | --- | --- | --- | --- |
| Region | 2014 | 2015 | 2016 | 2017 | 2018 | 2019 | Total |
| Changchun | 0 | 13 | 0 | 0 | 19 | 31 | 63 |
| Jilin | 5 | 0 | 7 | 45 | 30 | 0 | 87 |
| Baishan | 0 | 0 | 0 | 0 | 3 | 7 | 10 |
| Baicheng | 0 | 0 | 0 | 0 | 1 | 9 | 10 |
| Tonghua | 0 | 15 | 17 | 0 | 0 | 0 | 32 |
| Yanbian | 31 | 0 | 0 | 0 | 0 | 0 | 31 |
| Liaoyuan | 0 | 20 | 0 | 0 | 0 | 0 | 20 |
| Siping | 2 | 0 | 0 | 0 | 0 | 0 | 2 |
| Songyuan | 0 | 5 | 0 | 0 | 0 | 0 | 5 |
| Total | 38 | 48 | 24 | 45 | 53 | 47 | 260 |

**Table S4** Distribution of *Salmonella* isolates of frequently detected serotypes in each year

| Years(n) | The frequently detected *Salmonella* serotypes in each year (No. of isolates) | | | | | | |
| --- | --- | --- | --- | --- | --- | --- | --- |
|  | 2014(n=38) | 2015(n=52) | 2016(n=23） | 2017(n=45) | 2018(n=51) | 2019(n=44) | Total(n=253) |
| No. of *Salmonella*  serotype | 5 | 6 | 4 | 9 | 9 | 7 | 17 |
| *S*. Enteritidis | 18 | 35 | 9 | 19 | 21 | 21 | 123 |
| *S*. 1,4,[5],12:i:- | 15 | 8 | 13 | 16 | 18 | 16 | 86 |
| *S*. Typhimurium | 2 | 3 | 1 | 4 | 5 | 4 | 19 |
| *S*. Agona | 2 | 4 | 0 | 0 | 1 | 1 | 8 |
| *S*. Stanley | 0 | 2 | 0 | 1 | 1 | 1 | 5 |
| *S*. Litchfield | 0 | 1 | 0 | 0 | 2 | 0 | 3 |
| *S*. London | 0 | 0 | 0 | 0 | 1 | 2 | 3 |
| *S*. Thompson | 0 | 0 | 0 | 1 | 1 | 0 | 2 |
| *S*. I 4:b:- | 0 | 0 | 0 | 0 | 2 | 0 | 2 |
| *S*. Virchow | 0 | 0 | 0 | 1 | 0 | 0 | 1 |
| *S*. Chailey | 0 | 0 | 0 | 0 | 0 | 1 | 1 |
| *S*. Braenderup | 0 | 0 | 0 | 1 | 0 | 0 | 1 |
| *S*. Singapore | 0 | 0 | 0 | 1 | 0 | 0 | 1 |
| *S*. Havana | 0 | 0 | 0 | 1 | 0 | 0 | 1 |
| *S*. Derby | 1 | 0 | 0 | 0 | 0 | 0 | 1 |
| *S*. Infants | 0 | 0 | 1 | 0 | 0 | 0 | 1 |
| Untypable | 0 | 0 | 0 | 0 | 1 | 1 | 2 |

**Figure S1** A minimum spanning tree of *Salmonella* based on MLST displayed by cities sources. The circle size represents the number of strains. STs were labeled in circles and distinguished by color. Gray shadows between strains hint the close relationships between strains.

**
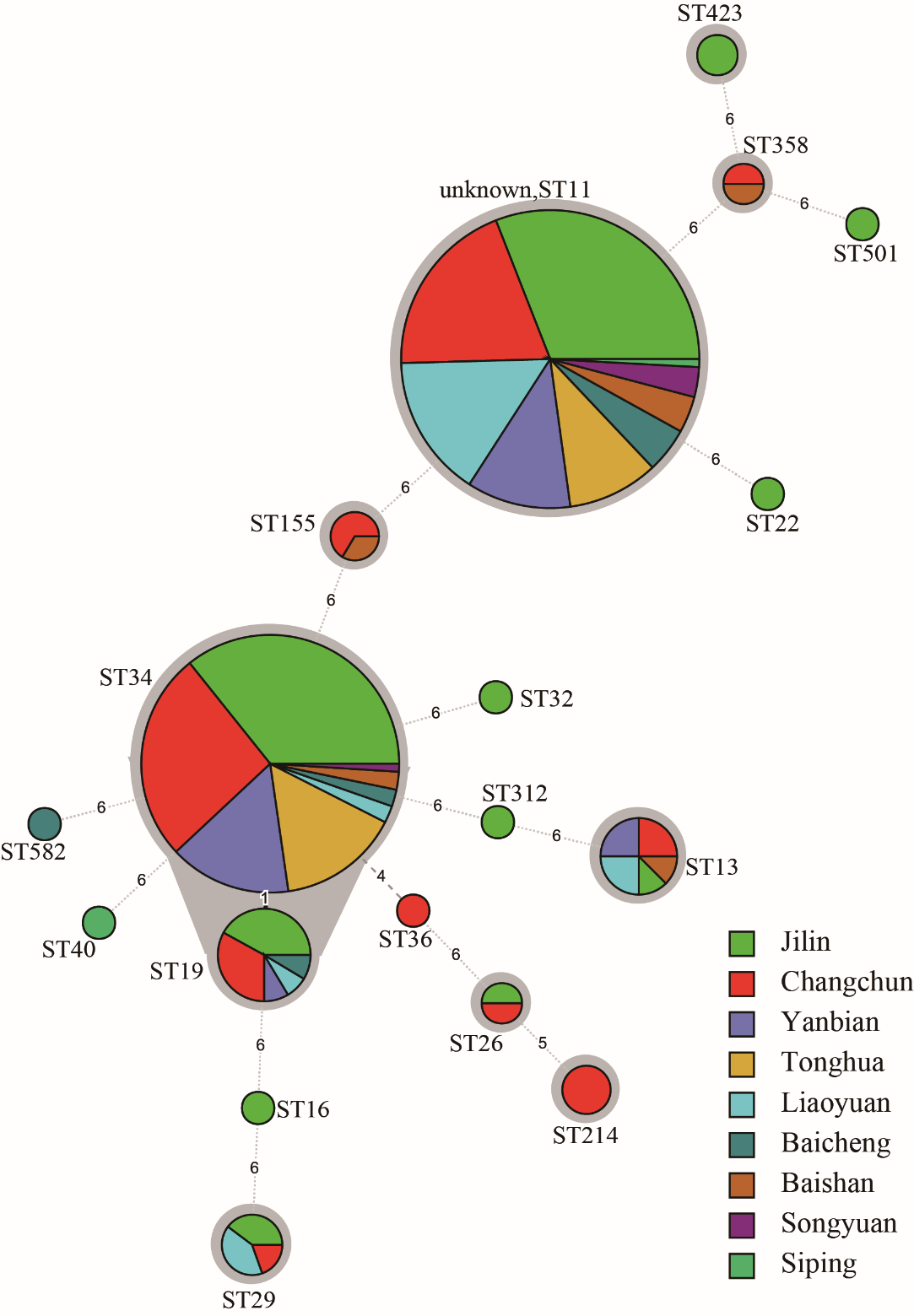
**

**Figure S2** A minimum spanning tree of *Salmonella* based on cgMLST displayed by cities sources. The circle size represents the number of strains. STs were labeled in circles and distinguished by color. Gray shadows between strains hint the close relationship between strains.

**
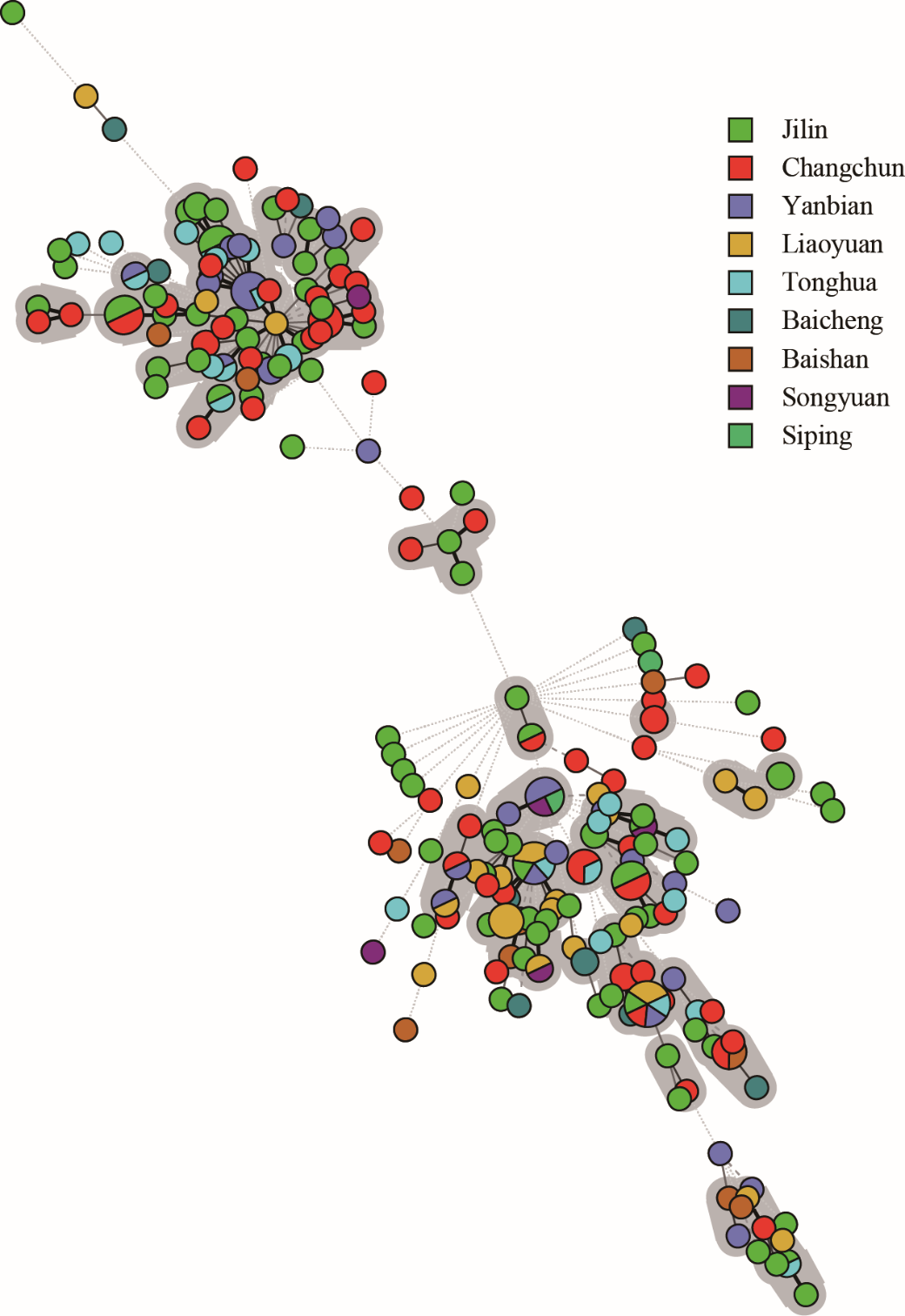
**

**Figure S3** Dendrogram of 92 *Salmonella* isolates of cluster A constructed based on cgMLST. Strain names were showed along with isolate city, year, STs.

**
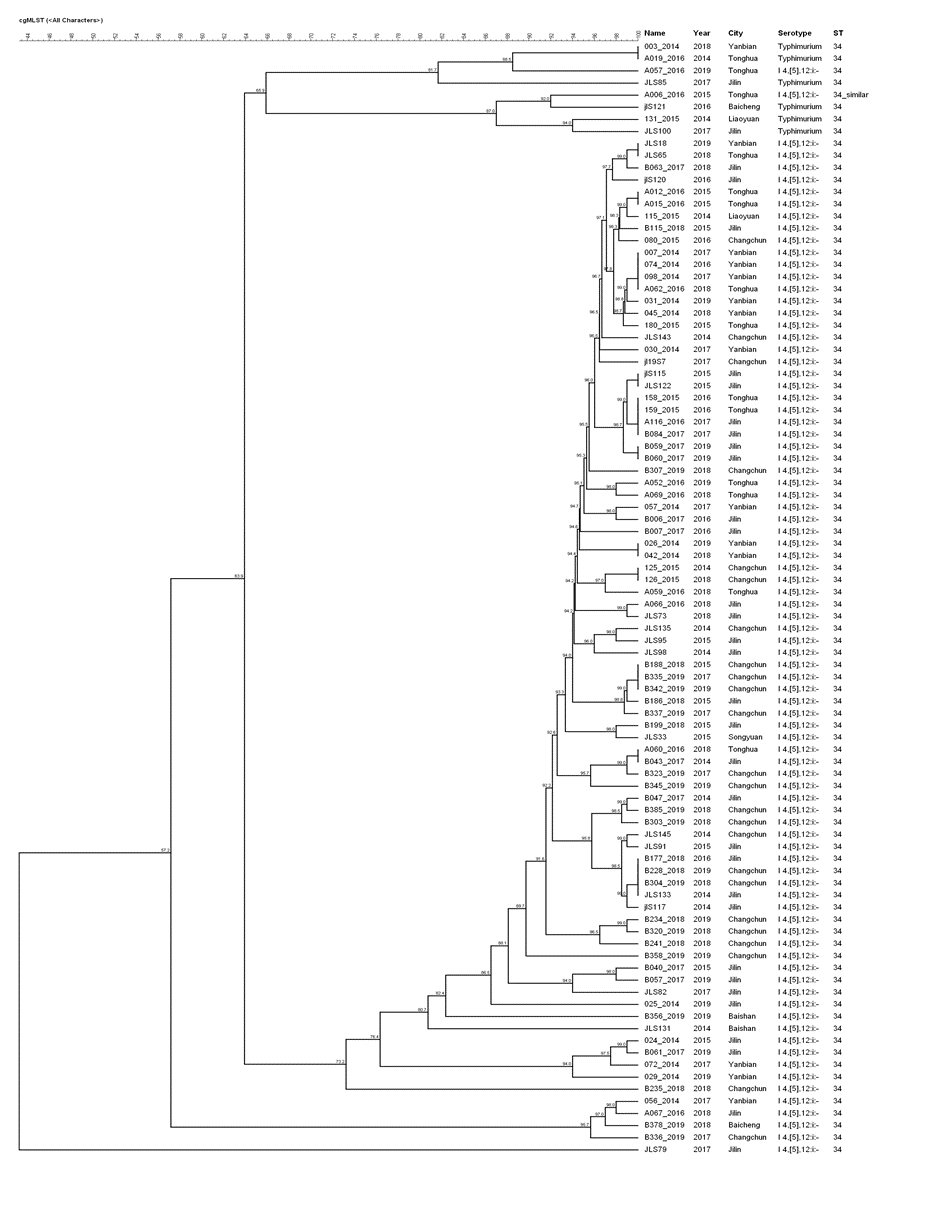
**

**Figure S4** Dendrogram of 9 *Salmonella* isolates of cluster B constructed based on cgMLST. Strain names were indicated along with isolate city, year, STs.

**
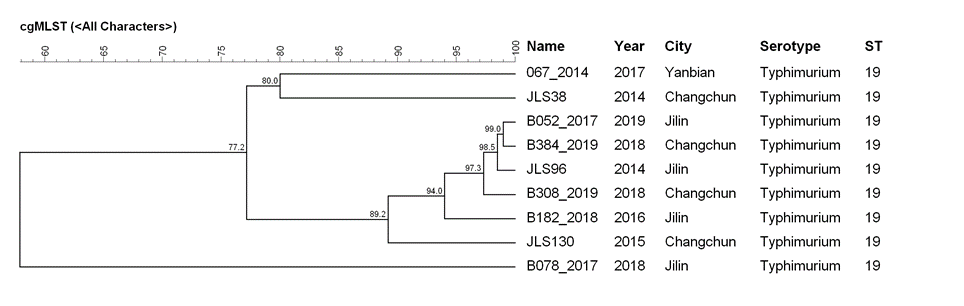
**

**Figure S5** Dendrogram of 112 *Salmonella* isolates of cluster C constructed based on cgMLST. Strains names were indicated along with isolate city, year, STs.

**
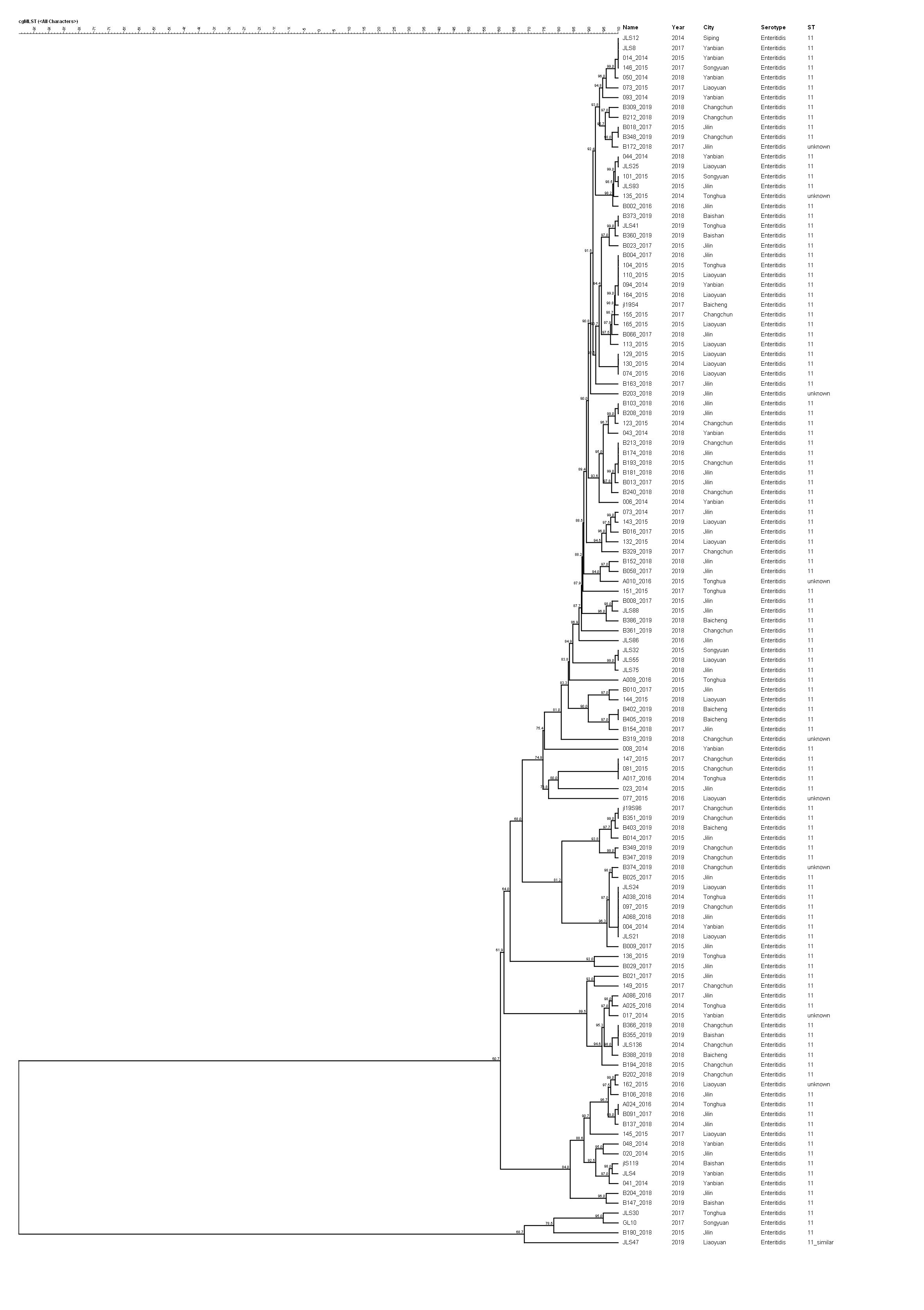
**
